# Human Bone Marrow-Derived Stem Cells Acquire Epithelial Characteristics through Fusion with Gastrointestinal Epithelial Cells

**DOI:** 10.1371/journal.pone.0019569

**Published:** 2011-05-05

**Authors:** Jonathan Ferrand, Danièle Noël, Philippe Lehours, Martina Prochazkova-Carlotti, Lucie Chambonnier, Armelle Ménard, Francis Mégraud, Christine Varon

**Affiliations:** 1 Laboratoire de Bactériologie, Université de Bordeaux, Bordeaux, France; 2 INSERM, U853, Bordeaux, France; 3 Université de Montpellier 1, Montpellier, France; 4 INSERM, U844, Montpellier, France; 5 EA2406, Université de Bordeaux, Bordeaux, France; Cardiff University, United Kingdom

## Abstract

Bone marrow-derived mesenchymal stem cells (MSC) have the ability to differentiate into a variety of cell types and are a potential source for epithelial tissue repair. Several studies have demonstrated their ability to repopulate the gastrointestinal tract (GIT) in bone marrow transplanted patients or in animal models of gastrointestinal carcinogenesis where they were the source of epithelial cancers. However, mechanism of MSC epithelial differentiation still remains unclear and controversial with trans-differentiation or fusion events being evoked. This study aimed to investigate the ability of MSC to acquire epithelial characteristics in the particular context of the gastrointestinal epithelium and to evaluate the role of cell fusion in this process. *In vitro* coculture experiments were performed with three gastrointestinal epithelial cell lines and MSC originating from two patients. After an 8 day coculture, MSC expressed epithelial markers. Use of a semi-permeable insert did not reproduce this effect, suggesting importance of cell contacts. Tagged cells coculture or FISH on gender-mismatched cells revealed clearly that epithelial differentiation resulted from cellular fusion events, while expression of mesenchymal markers on fused cells decreased over time. *In vivo* cell xenograft in immunodeficient mice confirmed fusion of MSC with gastrointestinal epithelial cells and self-renewal abilities of these fused cells. In conclusion, our results indicate that fusion could be the predominant mechanism by which human MSC may acquire epithelial characteristics when in close contact with epithelial cells from gastrointestinal origin . These results could contribute to a better understanding of the cellular and molecular mechanisms allowing MSC engraftment into the GIT epithelium.

## Introduction

Epithelial homeostasis, corresponding to a balance between epithelial cell loss and epithelial cell production, has to be maintained, particularly in the gastrointestinal tract (GIT) where the epithelium needs to be renewed extremely rapidly to ensure its function. The physiologic turnover of the epithelium is usually considered to be initiated by local progenitor cells in each gland which can give rise to different specialized epithelial cells [1]. However, mechanisms involved in tissue repair after damage remain poorly understood. Cells originating from extra-gastrointestinal sites and particularly from the bone marrow may take part in the repopulation of epithelial mucosa. Studies on gender-mismatched human bone marrow transplants showed that donor-derived cells can be retrieved in the epithelium of the gastrointestinal tract (GIT) [2]–[6]. Tissue damage, especially in grafts versus host disease or gastric ulcer, enhances engraftment of bone marrow-derived cells (BMDC), revealing a close relationship with the course of tissue regeneration [4]–[6]. The role of BMDC in epithelium repair was confirmed in mouse GIT as they can repopulate it in correlation with the level of damage after local irradiation or gastritis induced by chronic infection with *Helicobacter felis* [7]–[10].

From the different cell types constituting the stem cell population of bone marrow, mesenchymal stem cells (MSC) have been implicated in wound repair of numerous tissues [11]. MSC are multipotent cells, able to migrate across tissues to differentiate into a variety of cell types depending on the surrounding microenvironment. Among them, the best studied and characterized cell types both *in vivo* and *in vitro* are adipocytes, chondrocytes, osteoblasts and vascular smooth muscle cells [12]. In addition to these mesenchymal lineages, MSC have been reported to give rise to other cell types including epithelial cells [13]. However, due to the lack of good *in vitro* models, mechanisms of MSC epithelial differentiation and wound repair remain poorly understood and the few data available are controversial.

First, MSC may acquire epithelial characteristics by reprogrammation. *In vitro* studies showed that paracrine mechanisms, such as indirect coculture with lung epithelial cells or use of growth factors, allowed MSC differentiation in epithelial cells [14], [15]. MSC preparations do not appear to be homogeneous due to the lack of standardized protocols [16]. A study showed that MSC subsets expressing cytokeratin 19 were involved in the establishment of gastric epithelium after injection in mice [17]. These findings were confirmed *in vivo* as BMDC could acquire an epithelial phenotype in skin, liver and GIT without identification of any fusion event [8], [18], [19].

However, very few studies concluded that MSC differentiation occurs exclusively through a trans-differentiation mechanism, as fusion events were not really evaluated and cannot be excluded [20]–[22]. For example, indirect coculture with renal epithelial cells leads to infrequent MSC epithelial differentiation, whereas direct coculture leads to a greater percentage of differentiated cells [21]. The MSC adoption of different phenotypes may depend on the nature of physical interaction as MSC direct contact cocultured with keratinocytes expressed an epithelial phenotype in contrast to observations for noncontact cocultures [23]. Thus, acquisition of epithelial characteristics by MSC could be the consequence of fusion with epithelial cells. *In vitro* studies showed that MSC can fuse with small airway or lung epithelial cells thus leading to differentiated epithelial cells [15], [24]. Rizvi *et al.* demonstrated that BMDC may adopt the phenotype of intestinal cells *in vivo* by fusion with local epithelial cells after irradiation or in the context of intestinal cancer development [10].

Besides their role in wound repair, MSC may take part in carcinogenesis as accumulating data indicate that they can transform into malignant cells [8], [25], [26]. Due to their mesodermal origin, transformed MSC were shown to be at the origin of mesenchymal cancers such as Ewing's sarcoma and undifferentiated sarcoma in aging mice [25], [26]. The ability of MSC to differentiate into cells of endodermal origin suggests that MSC could be the source of other types of cancers. Concerning the GIT, MSC were described to be at the origin of gastric carcinoma or pre-neoplasic dysplasia in Barrett's esophagus [8], [27]. In addition, the presence of MSC in the tumoral environment could also favor cancer development by differentiation into tumor-associated fibroblasts contributing to angiogenesis and metastasis formation [28]–[30], although this role remains controversial [31]–[33].

In order to better understand the role of MSC in GIT epithelium physiopathology, this study aimed to evaluate the mechanisms by which human bone marrow-derived MSC cultured with epithelial cells may acquire epithelial characteristics *in vitro*. This may further allow a better understanding of the mechanisms of MSC engraftment into the GIT epithelium and their participation in gastrointestinal physiology and diseases.

## Results

### Characterization of MSC

**Figure 1:**
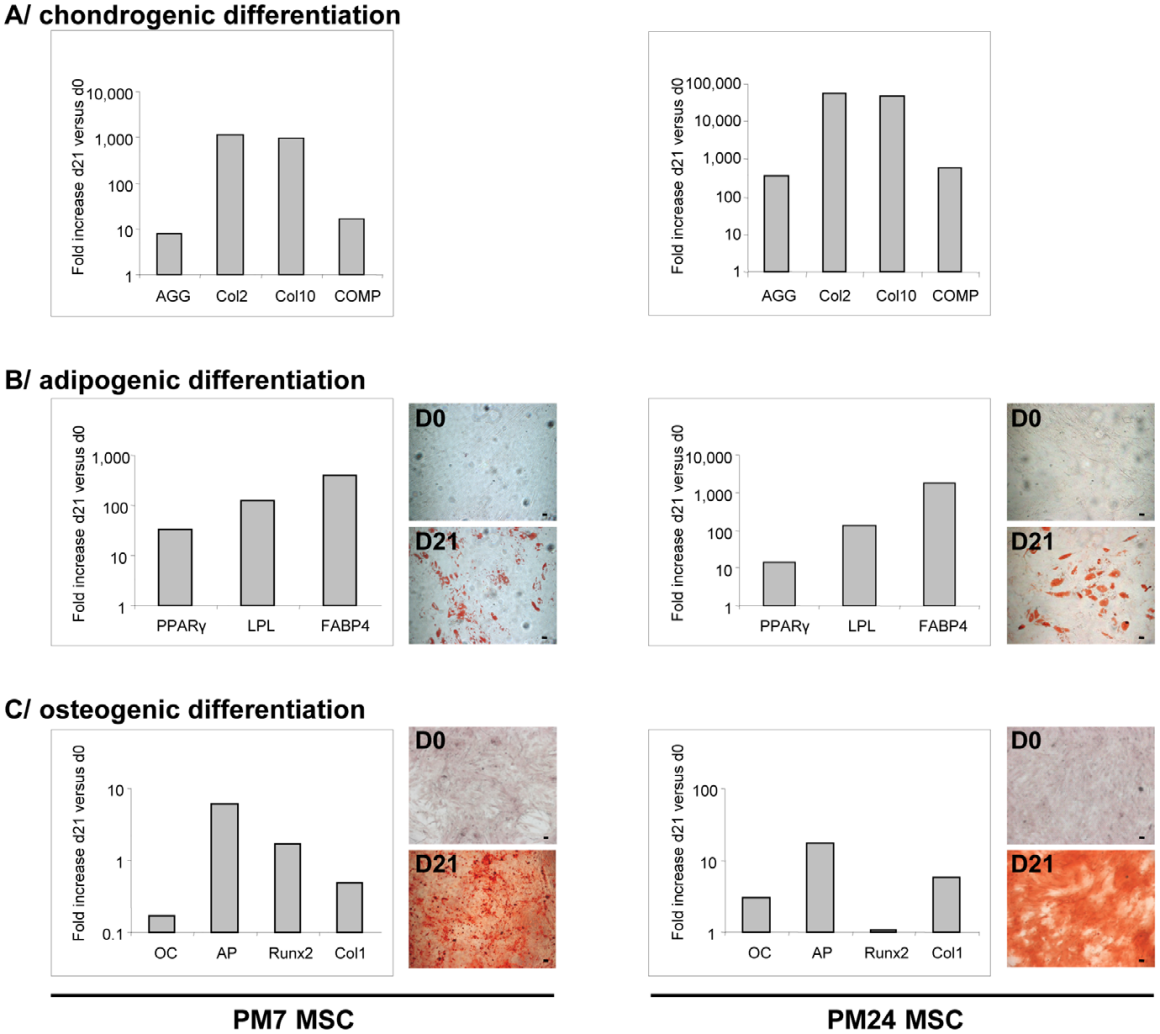
Trilineage differentiation of MSC. Left panels show differentiation of PM7 and right panels show that of PM24. **A/** Chondrogenic differentiation was visualized by detection of AGG, Col2, Col10 and COMP mRNAs using RT-PCR. **B/** Adipogenic differentiation was visualized by PPAR γ, LPL and FABP4 mRNAs using RT-PCR and Oil Red O staining of lipid droplets on day 0 **(D0)** and 21 **(D21)**. **C/** Osteogenic differentiation was visualized by OC, AP, Runx2 and Col1 mRNAs using RT-PCR and Alizarin Red S staining on day 0 **(D0)** and 21 **(D21)**. RT-PCR were performed on day 21 and results were compared to undifferentiated MSC on day 0 and normalized with RSP9 mRNAs. One representative experiment out of three is presented. Original magnification ×50. Scale bar, 10 µm.

After isolation from two different human donors who underwent hip replacement for osteonecrosis, cultured BMDC exhibited fibroblast-like cell morphology, typical phenotypes and possessed self renewal properties. PM7 and PM24 cells were positive for CD73, CD90, CD105 and negative for CD34, CD45, CD11b and CD14 (data not shown). After chondrogenic induction, AGG, Col2, Col10 and COMP mRNAs were increased compared to non-induced MSC (Figure 1A). When cultured in an adipogenic medium, expression of PPAR γ, LPL and FABP4 mRNAs was increased. In addition, differentiated cells displayed a positive staining of cytoplasmic lipid droplets with oil red O (Figure 1B). After 21 days of osteogenic differentiation, a slight increase of Runx2 mRNA, which is a early marker of osteogenesis, was observed. All the other osteoblastic markers tested (OC, AP, Runx2 and Col1) were up-regulated. More importantly, secretion of a mineralized matrix, as shown by positive alizarin red S staining, was observed demonstrating differentiation of MSC towards osteoblasts.(Figure 1C).

The two cell populations obtained, PM7 and PM24, fulfilled the criteria proposed by the Mesenchymal and Tissue Stem Cell Committee of the International Society for Cellular Therapy to define MSC [34].

### Human MSC may express epithelial markers after coculture with gastrointestinal cell lines

**Figure 2:**
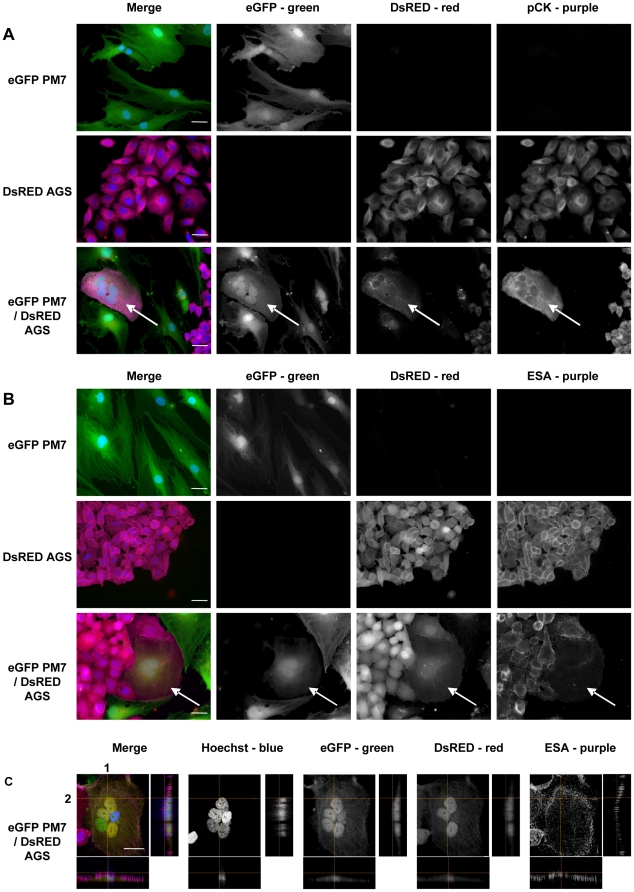
Immunofluorescent staining of epithelial markers in MSC cocultured with gastric epithelial cells *in vitro*. eGFP MSC (PM7) cells were cocultured with DsRED AGS epithelial cells for 8 days. **A/** Immunostaining with cytokeratins (pCK) or **B/** ESA primary antibodies were revealed with AlexaFluor 647 labelled secondary antibodies (purple), and nuclei were stained with Hoechst 33342 compound (blue). **C/** Three-dimensional reconstruction of confocal laser microscopy imaging. Images shown represent maximum intensity projection on the *x–y* axis of the *z*-stack and the projections of the orthogonal sections (1 and 2 dotted white lines) of the *z*-stack at the right side of each image. The first vertical panel shows colored merge images with Hoechst, whereas black and white channels alone follow. White arrows show MSC fused with epithelial cells and expressing cytokeratins. One representative experiment out of three is presented. Scale bar, 10 µm.

In order to study the ability of MSC to differentiate into gastric epithelial cells, GFP PM7 were cocultured with DsRED AGS gastric epithelial cells at a ratio of 8∶1 in culture plates (Figure 2). The 8∶1 ratio was chosen because of the difference of growth kinetics between MSC (low growth) and epithelial cell lines (rapid growth). After 8 days of coculture, cells expressing both eGFP and DsRED were detected (Figure 2A–B, white arrows). The presence of two or more nuclei was detected in most of these cells. As seen in the previous experiments, these heterokaryons were probably the consequence of plasma membrane fusion between the two cell populations. In addition, these fused cells were positive for epithelial cytokeratins, whereas MSC alone were negative (Figure 2A). In order to confirm the epithelial phenotype of fused cells, the expression of another specific epithelial marker, ESA, was evaluated. ESA was expressed at the cell surface of epithelial cells but not on the MSC (Figure 2B). eGFP and DsRED double positive cells expressed ESA (Figure 2B, white arrows).

We confirmed by confocal microscopy and 3D reconstitution that cells expressing eGFP, DsRED and ESA were single cells, as shown by the 3 labellings on the same z-level (Figure 2C).

Similar experiments were performed by direct coculture of eGFP PM7 with the DsRED transduced colon carcinoma cell line HT-29. After coculture, eGFP and DsRED double positive cells appeared, and these cells were positive for cytokeratins and ESA (Figure S1).

The same results were obtained using PM24 MSC (data not shown). We also verified that cell transduction was not responsible for an increased capacity of cells to fuse. MSC and epithelial cells were labelled green or red with membrane fluorescent dyes, PKH2 or CM-Dil compounds, respectively. PKH2-labelled MSC were positive for ESA and CM-Dil after 8 days of coculture with CM-Dil- labelled AGS, HT-29 or non-malignant gastric epithelial cells HFE-145 confirming the previous results (data not shown).

### Human MSC can acquire epithelial characteristics by a mechanism of cellular fusion with gastrointestinal epithelial cells

**Figure 3:**
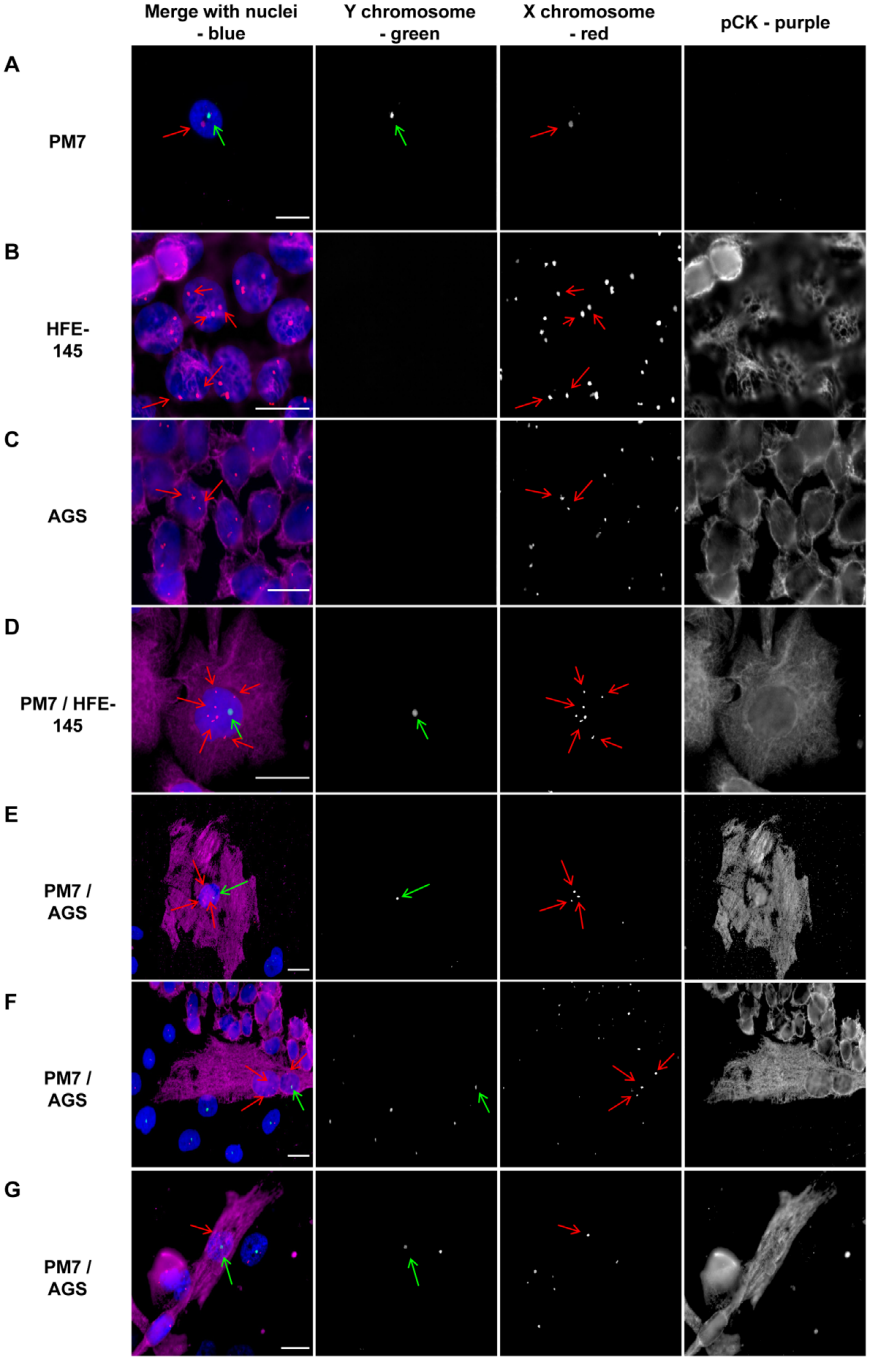
FISH analysis of MSC and epithelial gastric cell line cocultures. eGFP male MSC (PM7) cells were cultured with DsRED female epithelial AGS or HFE-145 cells for 8 days and fixed. FISH (SpectrumGreen-Y chromosome and SpectrumOrange-X chromosome) was performed and pancytokeratin primary antibodies were revealed with AlexaFluor 647 labelled secondary antibodies (purple), and nuclei were stained with Hoechst 33342 compound (blue). The first vertical panel shows colored merge images with Hoechst, whereas black and white channels alone follow. Green arrows show Y chromosomes and red arrows X chromosomes. **A–C/** Male PM7 expressed one Y chromosome and one X chromosome whereas female AGS or HFE-145 cells expressed only X chromosomes and cytokeratins. **D–E/** In PM7/HFE-145 or AGS cocultured cells: example of one cell expressing cytokeratins and possessing a Y chromosome corresponding to a MSC-derived cell. In PM7/AGS cocultured cells, examples of one cell **F/** expressing cytokeratins and harboring one male and one female nuclei or **G/** expressing cytokeratins and harboring one nucleus with one Y chomosome and one X chromosome. One representative experiment out of three is presented. Scale bar, 10 µm.

In order to confirm the MSC epithelial differentiation potential and to assess the role of fusion, coculture of gender-mismatched MSC and HFE-145 or AGS cells was performed, and phenotypes were evaluated by pancytokeratin immunostaining combined with FISH on sex chromosomes. Male PM7 and female AGS cells had normal sex chromosome content (Figure 3A, C). HFE-145 epithelial cells displayed female specific chromosome content but more than two X chromosomes were detected in many cells (Figure 3B). Both HFE-145 and AGS cells expressed epithelial pancytokeratin whereas MSC did not (Figure 3A–C).

An 8 day coculture led to the appearance of male-derived cells which were positive for pancytokeratin (Figure 3D–E). Some of the cells contained a single nucleus with abnormal content (ie one Y chromosome and several X chromosomes) indicating that the nucleus of a male MSC had fused with the nucleus of a female epithelial cell (Figure 3D–E). These results were observed both with HFE-145 and AGS cells. Detailed analysis of cocultured PM7 with AGS cells revealed that some of the pancytokeratin positive cells contained two nuclei, one with one X and one Y chromosome (from a male MSC) and the other with two X chromosomes (from a female AGS cell), indicating a fusion of membranes between a male MSC and a female epithelial cell without fusion of the two nuclei (Figure 3F). Finally, some cells expressed pancytokeratin and contained only one nucleus with a male content (one X and one Y chromosome), suggesting mitosis of fused multinucleated cells leading to PM7-derived mononucleated daughter cells with epithelial characteristics or PM7 epithelial differentiation without fusion events (Figure 3G).

### Fusion of human MSC and gastrointestinal cells leads to an epithelial phenotype at a relatively important frequency *in vitro*

**Figure 4:**
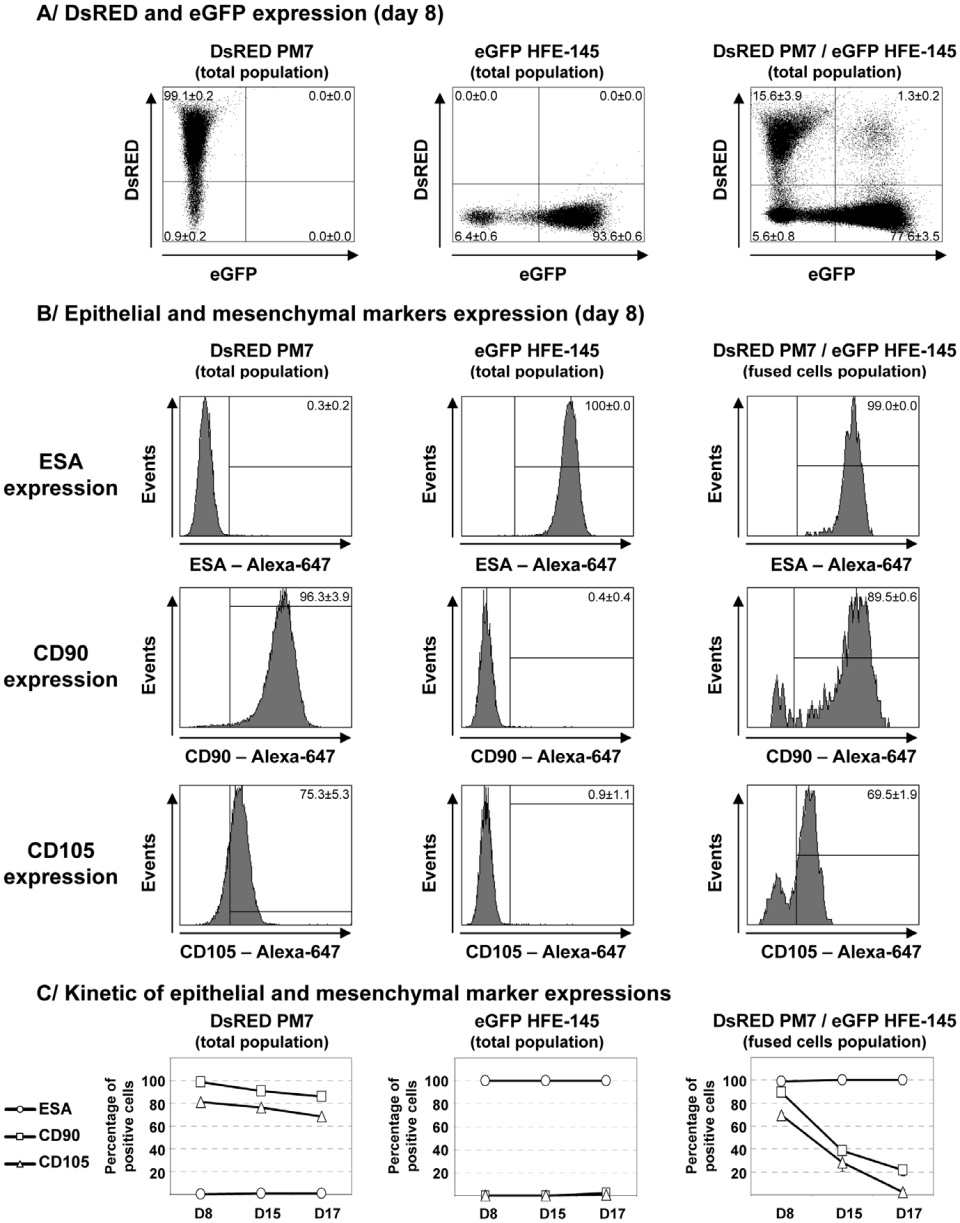
Quantification of fused MSC by flow cytometry. DsRED MSC (PM7) cells were cultured with eGFP epithelial HFE-145 cells for indicated times, harvested, stained with anti-ESA antibodies, detected by Alexa-647 labelled secondary antibodies and analyzed by flow cytometry. **A/** DsRED and eGFP expression of PM7, HFE-145 and cocultured cells was analyzed at day 9. Coculture of DsRED PM7 and eGFP HFE-145 cells led to the appearance of fused cells (1.3%±0.2 of total cells). **B/** Fused cells expression of epithelial (ESA) and mesenchymal (CD90 and CD105) markers was measured at day 9. Fused cells expressed ESA (99%±0.0 of positive cells); CD90 (89.5%±0.6) and CD105 (69.5%±1.9). **C/** Expression of epithelial and mesenchymal markers was assessed at day 9, 15 and 17. Expression of ESA was stable whereas expression of mesenchymal markers decreased over time. Results represent the mean ± SD of one experiment with three replicates representative of at least three different experiments.

To evaluate the frequency of MSC fusion with epithelial cells, DsRED PM7 and eGFP HFE-145 cells were cocultured for 8 days, harvested, immunostained for ESA as an epithelial marker and CD90 or CD105 as mesenchymal markers and analyzed by flow cytometry (Figure 4A). After an 8 day coculture, fused cells expressing both eGFP and DsRED appeared, while most of the cells were eGFP positive due to the rapid growth of epithelial cells compared to MSC (Figure 4A, right panel). The fused cells population counted 1.3±0.2% among total cells corresponding to 7.7±1.3% of DsRED positive cells. Using DsRED AGS and eGFP PM7 or DsRED HT-29 cells and eGFP PM24 MSC, 5.9±1.0% or 7.1±3.7% of MSC-derived cells expressed DsRED and ESA respectively confirming the results previously obtained with PM7 and HFE-145 cells (Table 1).

**Table 1:** Quantification of fusion events between MSC and epithelial cells by flow cytometry analysis.

Direct coculture	Percentage of eGFP+, DsRED+ and ESA+ cells
eGFP or DsRED PM7 alone	0.0±0.0%
DsRED PM7/eGFP HFE-145	7.7±1.3%
eGFP PM7/DsRED AGS	5.9±1.0%
eGFP PM7/DsRED HT-29	7.1±3.7%

Indirect coculture	Percentage of ESA+ MSC-derived cells
PM7 alone	0.23±0.05%
PM7/AGS	0.17±0.05%

For direct coculture, eGFP or DsRED MSC (PM7) cells were cultured with epithelial (DsRED AGS, eGFP HFE-145 and DsRED HT-29) cells for 8 days, harvested, stained with anti-ESA antibodies, detected by Alexa-647 labelled secondary antibodies. For indirect coculture, PM7 and epithelial cells (AGS) were separated by a 0.4 µm cell culture insert and processed as for direct coculture. Cells were then analyzed by flow cytometry. Results represent the mean ± SD of one experiment with three replicates representative of at least three different experiments.

Expression of ESA, CD90 and CD105 was then measured on fused cells to determine their epithelial or mesenchymal phenotype (Figure 4B). PM7 were negative for ESA staining but positive for CD90 and CD105 expression, while HFE-145 cells were positive for ESA and negative for CD90 and CD105 expression. Fused cells expressed ESA (99±0.0%) and heterogeneously CD90 and CD105 (89.5±0.6% and 69.5±1.9%, respectively) after an 8 days coculture. Expression of epithelial and mesenchymal markers was then measured over time (Figure 4C). Expression of ESA remained stable up to 17 days of coculture (100%±0.0, p = 1 versus day 8) whereas expression of CD90 and CD105 significantly decreased over time (21.5±4.8%, p<0.01 and 2.4±2.4%, p<0.01, respectively, at day 17 versus day 8). These results suggest that fused cells may loose their mesenchymal phenotype in favour of an epithelial phenotype over time.

Fused cells observed by immunofluorescent staining appeared larger than non fused cells (Figure 2). In order to quantify this observation, forward scatter was measured by flow cytometry. Over time, fused cells conserved a larger size compared to PM7 and HFE-145 cells (fused cells FSC-mean was 1.35±0.10 times higher than PM7 (p<0.01) and 1.85±0.14 higher than HFE-145 (p<0.01) at day 17), confirming these findings.

Fusion appeared to be the predominant mechanism allowing expression of epithelial markers by PM7 as only 15.3±0.6% of ESA-positive DsRED PM7 were negative for eGFP expression at day 8 and 6.8±1.0% at day 17. These cells possibly correspond to MSC fused with non-transduced epithelial cells or trans-differentiated cells or a loss of eGFP gene in a fused cell after mitosis event (data not shown). In order to determine the ability of PM7 to acquire epithelial characteristics without fusion events, eGFP PM7 and DsRED AGS cells were cocultured of both sides of a 0.4 µm-cell culture insert. After indirect coculture for 8 days, 0.17±0.05% of PM7 expressed ESA staining versus 0.23±0.05% when PM7 were cultured alone (p = 0.23), strongly excluding the ability of PM7 to trans-differentiate in epithelial cells (Table 1).

### Fusion of MSC and epithelial cells can be observed in *in vivo* system

**Figure 5:**
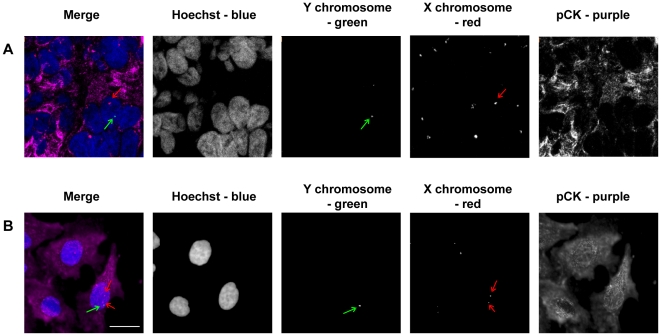
FISH analysis of chromosome content of tumor cells resulting from human MSC and AGS cell xenografts in immunodeficient mice. **A/** Male MSC (PM7) and female AGS cells were injected subcutaneously in NOG mice. After 50 days, mice were sacrificed and tumors resulting from transplanted cells were processed and analyzed by immunofluorescence and FISH (SpectrumGreen-Y chromosome and SpectrumOrange-X chromosome). Cytokeratins were stained with primary antibodies revealed by secondary AlexaFluor 647 labelled antibodies (purple), and nuclei were stained with Hoechst 33342 compound (blue). The first vertical panel shows colored merge images, whereas black and white channels alone follow. Red and green arrows show chromosomes of one male-derived cell expressing cytokeratins. **B/** Cells from tumors were dissociated and cultured *in vitro* for 24 h. Cells were processed as in A. Male derived cells expressing cytokeratins possess abnormal chromosome content but are viable and adhere to coverslips. White bar, 10 µm.

AGS cells mixed freshly with MSC (AGS∶MSC) at the same ratio as that used in *in vitro* assays (1∶8) were xenografted by subcutaneous injection in NOG immunodeficient mice. Fifty days post-injection, the mice were sacrificed and tumors were recovered for analysis. Rare MSC-derived epithelial cells displaying a nucleus with one Y chromosome and expressing cytokeratins were observed (Figure 5A). In addition, confocal microscopy revealed that some single cells had nuclei with abnormal content which may have resulted from a fusion event (nucleus with one Y and several X chromosomes) (data not shown). Moreover, when cells from fresh tumors were dissociated and cultured overnight on glass coverslips, fused cells were observed by nuclei contents analysis by FISH (Figure 5B). These results indicate that fusion events occurring *in vivo* led to viable cells able to adhere and survive *in vitro*.

## Discussion

In this study, we demonstrated for the first time that MSC can acquire epithelial characteristics through a fusion mechanism with gastric and intestinal epithelial cells. As primary gastrointestinal epithelial cells are not available or cultivable *in vitro* over a long period of time, two malignant (AGS and HT-29 cells) and one non-malignant (HFE-145 cells) human gastrointestinal epithelial cell lines were cocultured with two different samples of human MSC. The same results were obtained in cocultures of MSC with cancerous cells and non-cancerous cells as well as in an *in vivo* model, reinforcing the hypothesis of the fusion process identified by different techniques. Such evidence was lacking in previous studies in which fusion events were not investigated in depth [20]–[22]. Acquisition of an epithelial phenotype through cell fusion was confirmed by the protein expression of two specific epithelial markers not expressed by MSC alone, excluding a misleading gene expression as is sometimes the case in neural differentiation [35]. This process involves a loss of mesenchymal markers expression during time suggesting that the fused cells will evolve into epithelial cells. We hypothesize that fusion events observed *in vitro* could be a mechanism of engraftment leading to epithelial differentiation *in vivo*. Engrafted MSC, undergoing stimuli from microenvironment, may then be reprogrammed *in vivo*.

To date, it is well known that MSC are multipotent cells as they can differentiate into different mesenchymal lineages and participate to tissue physiopathology. In addition, MSC are frequently considered as pluripotent cells since they have been reported to give rise to cell types of neuroectodermal or endodermal origins, *e.g.* endothelial, skeletal and cardiac muscle, neural and epithelial cells; and hepatocytes [13]. The differentiation is easily demonstrated by using culture conditions specific for differentiation into adipocytes, chondrocytes, osteoblasts and vascular smooth muscle cells [36], but is more difficult to investigate when using *in vitro* coculture or *in vivo* systems to study differentiation into hepatocytes, myoblasts or epithelial cells [12], [17], [37], [38]. The models in our study clearly show that one mechanism of acquisition of epithelial characteristics by MSC is fusion events. The presence of MSC-derived cells was also confirmed by the use of FISH, lentiviral transduction and chemical staining. In addition, the model of direct coculture used in the present study could be considered to be closer in a way to the physiological context compared to indirect coculture using previously described cell culture insert systems, where trans-differentiation events were observed [15], [21]. In our study, cell culture inserts did not allow trans-differentiation events, nor did the use of growth factors as described by Paunescu *et al.* (data not shown) [14]. In order to determine the fate of the fused cells overtime, we considered it important to investigate this phenomenon in xenograft experiments *in vivo* using immunodeficient recipient mice. Fused cells were found *in vivo* at the time of sacrifice fifty days later and *in vitro* after culture of dissociated cells, indicating that they are able to survive *in vivo*.

Fusion between BMDC and other tissue specific cells has already been shown *in vitro* and *in vivo* with embryonic stem cells, myoblasts, hepatocytes in the liver, Purkinje neurons, cardiac muscle in the heart, and airway epithelial cells [15], [24], [37], [39], [40]. An exciting hypothesis is that in the context of gastrointestinal epithelia repair, the fusion of MSC with epithelial cells could be of major importance in initiating a rapid differentiation and an effective integration in the GIT during Helicobacter infection, gastric ulcer, esophagojejunostomy, graft versus host disease or irradiation [1], [7], [8], [10], [27].

FISH experiments on gender-mismatched cocultured cells showed that fusion also concerned nuclei. The observation of cells displaying a single nucleus with a normal sexual chromosome content and the absence of trans-differentiated cells suggest that a fusion event may be followed by normal mitosis. Fusion has already be shown to be responsible for nuclei reprogramming of differentiated cells resulting in mitosis and selection for survival [41]. Some authors even suggested that fusion events are an obligatory step in the occurrence of cancer cells to acquire self renewal and migratory properties [42], [43]. In a mouse model, complete units of dysplastic gastric epithelial glands were reconstituted of MSC [8]. Interestingly a recent study showed that the frequency of cellular fusion between MSC and cerebellar neurons increased in the presence of TNF-α and/or IFN-γ [44]. Considering our results, MSC fusion with gastrointestinal epithelial cells may involve a reprogramming of the fused cells rendering them more susceptible to transformation and leading to the appearance of cancer stem cells in response to a chronic stress like inflammation. Alternatively, MSC fusion with epithelial cells could promote the cancerous process rather than initiate it. The *in vitro* and *in vivo* models developed in the present study could pave the way to a better understanding of the role of MSC-derived epithelial cells in inflammation-related diseases and cancers.

In conclusion, we demonstrated that MSC and gastrointestinal epithelial cells can fuse when cultured in close contact both *in vitro* and *in vivo*, conferring epithelial characteristics to MSC. The models proposed will allow the study of the differentiation mechanisms, as well as the key transcription factors involved, the long term evolution of differentiated MSC and their potential role in carcinogenesis.

## Materials and Methods

### Ethics Statement

Approval was obtained from the French Committee of Genetic Enginneering (approval number 4608) and the local Central Animal Facility Committee of the University of Bordeaux before initiation of the study. All animal experiments were performed in level 2 animal facilities of University *Victor Segalen* Bordeaux 2, in accordance with institutional guidelines as determined by the Central Animal Facility Committee of the University and in conformity with the French Ministry of Agriculture Guidelines or Animal Care.

Consent of MSC donors was written and approved by the French Ministry of Research and the Languedoc Roussillon ethic committee (approval number DC2009-1052).

### Cell cultures

Human gastric carcinoma AGS cells (CRL-1739, ATCC, Molsheim, France) were cultured in Dulbecco's Modified Eagle Medium - nutrient mixture F12 (DMEM-F12) and colon carcinoma HT-29 cells (ACC 299, DSMZ collection, Braunschweig, Germany) in McCoy's 5A medium, supplemented with 10% fetal calf serum (FCS) and antibiotics (100 U/ml penicillin and 100 µg/ml streptomycin, all from Invitrogen, Cergy Pontoise, France). Human non-cancerous cell line HFE-145 was a kind gift from D. Smoot (Howard University, Washington, USA) and was cultured in the medium previously used for AGS cells [45].

MSC cultures were established from bone marrow samples of donors suffering from osteonecrosis, a disease linked to insufficient blood supply commonly associated with long-standing corticosteroid use, undergoing hip replacement [46]. Although it has been reported that the differentiation of mesenchymal stem cells to adipocytes may be one of the mechanisms causing increased intraosseous pressure and collapse of marrow sinusoids, autologous BM-MSCs have been proposed as a novel treatment option [47]. Cells were plated at a concentration of 5×10^4^ cells/cm^2^ in Minimal Essential Medium α (Invitrogen) supplemented with 10% FCS, 1 ng/ml basic fibroblast growth factor (bFGF; R&D Systems, Lille, France) and antibiotics. At subconfluence, cells were harvested and plated at 1 000 cells/cm^2^. Samples from two donors, an 82 year old man and a 66 year old woman, called PM7 and PM24 respectively, were used between passages 3 and 9.

### MSC characterization

Cell surface antigen expression of CD11b, CD34, CD45 (Beckman Coulter, Villepinte, France), CD14 (eBiosciences, San Diego, CA, USA), CD73, CD90 and CD105 (BD Biosciences, Le Pont de Claix, France) was measured by flow cytometry.

For chondrogenic differentiation, 2.5×10^5^ cells were centrifugated at 600 g for 5 min. The resulting pellets were cultured in DMEM supplemented with 0.1 µM dexamethasone, 0.17 mM ascorbate-2-phosphate, 1% insulin-transferrin-sodium selenite supplement (all from Sigma, l'Isle d'Abeau, France) and 10 ng/ml of recombinant Transforming Growth Factor β3 (R&D Systems).

For adipogenic differentiation, cells were plated at a density of 8×10^3^ cells/cm^2^ and cultured in DMEM containing 5% FCS (Invitrogen), 1 µM dexamethasone, 50 µM isobutyl-methylxanthine and 60 µM indomethacin (all from Sigma).

Osteogenesis was induced by culture at low density (2.5×10^3^ cells/cm^2^) in DMEM with 10% FCS, 10 mM β-glycerophosphate, 0.1 µM dexamethasone and 0.05 mM ascorbic acid (all from Sigma).

Media were changed three times a week. Differentiations were assessed on day 21 by real-time reverse transcription PCR (RT-qPCR) on extracted mRNAs, by visualization of lipid droplets after oil red O staining for adipogenesis and by visualization of matrix calcification after Alizarin red S staining for osteogenesis.

### RNA preparation and RT-qPCR

For chondrogenesis, micropellets were washed in PBS and mechanically dissociated. For adipogenis and osteogenis, cells were rinsed with PBS and the lysis buffer was added. Total RNA was extracted using the RNeasy Kit according to the manufacturer's recommendations (Qiagen S.A., Courtaboeuf, France). Chondrogenic differentiation was visualized by detection of aggregan (AGG), collagen type II (Col2), collagen type X (Col10) and cartilage oligomeric matrix protein (COMP) mRNAs; adipogenic differentiation by detection of peroxisome proliferator-activated receptor γ (PPAR γ), lipoprotein lipase (LPL) and fatty acid binding protein 4 (FABP4) mRNAs; and osteogenic differentiation by osteocalcin (OC), alkaline phosphatase (AP), Runx2 and collagen type I (Col1) mRNAs (personal data). RT-PCR was performed on day 21 and results were compared to undifferentiated MSC on day 0.

### Cellular labelling

The eGFP encoding TMEW and the DsRED encoding TMDW lentiviral vectors were kindly provided by F. Moreau Gaudry (Vectorology Platform, Université *Victor Segalen* Bordeaux 2, France). Gastrointestinal epithelial cells and MSC were transduced respectively with lentiviral particles containing the vectors encoding DsRED and eGFP at multiplicities of infection ranging from 1 to 20. Twenty-four hours after transduction, transduction efficiency was measured by flow cytometry and cell samples with 30% positive cells were sorted to obtain a homogeneous population.

For staining with fluorescent chemical compounds, the PKH2 Green Fluorescent Cell Linker Kit (Sigma) and CM-Dil cell labelling solution (Vybrant, Invitrogen) were used according to the manufacturer's recommendations. Briefly, cells were harvested and resuspended in 1 ml of labelling solutions (PKH2 or CM-Dil) for the indicated time in the manufacturer's instructions. Cells were washed three times in culture medium before use.

### Coculture experiments and fluorescent staining

Coculture experiments of MSC and epithelial cells were performed at a ratio of 8∶1 (20 000 MSC:2 500 epithelial cells) in 24-well culture plates. Cells were cultured on 12 mm glass coverslips for immunofluorescent stainings. A 0.4 µm cell culture insert system was used for the indirect coculture assay (BD Biosciences). 20 000 MSC and 2 500 epithelial cells were seeded on the bottom chamber or on the top chamber, respectively. Coculture experiments were performed for indicated times in MSC medium without bFGF changed three times a week.

For immunofluorescent staining, cells were washed with PBS to remove cellular debris, then fixed with 3% paraformaldehyde prepared in cytoskeletal buffer for 10 min and processed as previously described [48]. Primary and secondary antibodies were diluted at the following concentrations: 1∶100 for mouse anti-Epithelial Specific Antigen (ESA) antibodies (StemCell Technologies, Grenoble, France), 1∶100 for mouse anti-pancytokeratin antibodies (Ozyme, St Quentin Yvelines, France), 1∶500 for Alexa-647 labelled goat anti-mouse antibodies (Molecular Probes, Invitrogen) and Hoechst 33342 (1 µg/ml) compound was used as nuclear counterstain (Molecular Probes, Invitrogen). Coverslips were washed in water and mounted on microscope slides with Fluoromount mounting medium (Clinisciences SA, Montrouge, France).

For Fluorescence *In Situ* Hybridization (FISH), cells were fixed in 3.7% formaldehyde solution in PBS for 10 min, washed in PBS and dehydrated in ethanol series before hybridization. FISH experiments with the alpha satellite centromeric region of the X chromosome and the satellite III (Yq12) region of the Y chromosome were performed according to manufacturer's instructions (Abbott Molecular, Rungis, France). Coverslips were washed in PBS, and immunofluorescently stained as previously described.

For flow cytometry analysis, cells were incubated at room temperature with mouse anti-ESA antibodies, rat anti-CD90 or rat anti-CD105 in PBS (1∶100) for 20 min, washed and incubated with Alexa-647 labelled anti-mouse or anti-rat secondary antibodies in PBS (1∶200) for 15 min. Cells were resuspended in PBS before being analyzed in a BD FACSCanto II flow cytometer using FACSDiva software (BD Biosciences).

### Mouse *in vivo* xenografts

Immunodeficient non obese diabetous/Shi-severe combined immunodeficiency/interleukin-2Rγ^null^ (NOG) mice were injected with PM7 and AGS mixed at a ratio of 8∶1 (2 250 000 and 375 000 cells, respectively) resuspended in 200 µl of 7 mg/ml Matrigel (BD Biosciences) in ice cold PBS. Mice were anesthetized with 3% isofluran (Belamont, Boulogne Billancourt, France) before subcutaneous injection into the right flank. When tumor sizes reached 100 mm^2^, mice were sacrificed by cervical dislocation and tumors immediately harvested, embedded in Optimal Cutting Temperature (OCT) compound (Sakura, Labonord, Villeneuve d'Asq, France) and snap frozen in cooled isopentane with liquid nitrogen. Tissues were stored at −80°C before being cut into 7 µm thick serial sections on a cryostat (Leica Microsystems, Nanterre, France). Tissue sections mounted on glass slides were processed as previously described.

Some tumor samples were cultured on glass coverslips after dissociation. Briefly, after mechanical mincing, samples were dissociated by incubation with 0.1% collagenase and 0.0125% hyaluronidase in PBS (all from Sigma) for 45 min at 37°C and filtered through a 70 µm cell strainer. Erythrocytes were lysed at 4°C for 10 min with 170 mM NH_4_Cl, 2 mM KHCO_3_ and 0.1 mM ethylene-diamine-tetra-acetic acid (EDTA) before plating the cells on glass coverslips. After an overnight culture, cells were fixed with 3.7% formaldehyde and processed for stainings.

### Microscopy analysis

Cells were analyzed using a Eclipse 50*i* epi-fluorescence microscope (Nikon, Champigny sur Marne, France) equipped with the Nis Element acquisition software and a ×40 (numerical aperture, 1.3) oil immersion objective or a DMI6000 confocal microscope (Leica Microsystems, Rueil Malmaison, France) equipped with the LEICA acquisition software and a ×63 (numerical aperture, 1.4) oil immersion objective. Z-stack acquisitions were performed by 0.5 µm slicing.

### Statistical analysis

Quantification values represent the means of one experiment with three replicates representative of at least three different experiments in each ± standard deviation (SD). For immunofluorescent microscopy analysis, a minimum of 500 cells were analyzed and one representative experiment out of three is presented. For flow cytometry analysis, 100,000 cells were analyzed for each condition to determine the mean percentage of positive cells for studied parameters. Significance was determined using the Student's t-test.

## Supporting Information

Figure S1 **Immunofluorescent staining of epithelial markers in MSC cocultured with colon epithelial cells** ***in vitro*** **.** eGFP MSC (PM7) cells were cocultured with DsRED HT-29 epithelial cells for 8 days. **A/** Immunostaining with cytokeratins (pCK) or **B/** ESA primary antibodies were revealed with AlexaFluor 647 labelled secondary antibodies (purple), and nuclei were stained with Hoechst 33342 compound (blue). The first vertical panel shows colored merge images with Hoechst, whereas black and white channels alone follow. White arrows show MSC fused with epithelial cells and expressing cytokeratins. One representative experiment out of three is presented. Scale bar, 10 µm.
(TIF)
