# Supplementary figures and images for: Human Bone Marrow-Derived Stem Cells Acquire Epithelial Characteristics through Fusion with Gastrointestinal Epithelial Cells
Source: PLoS One. 2011 May 5;6(5):e19569. doi: 10.1371/journal.pone.0019569 (PMC3088703; doi:10.1371/journal.pone.0019569)

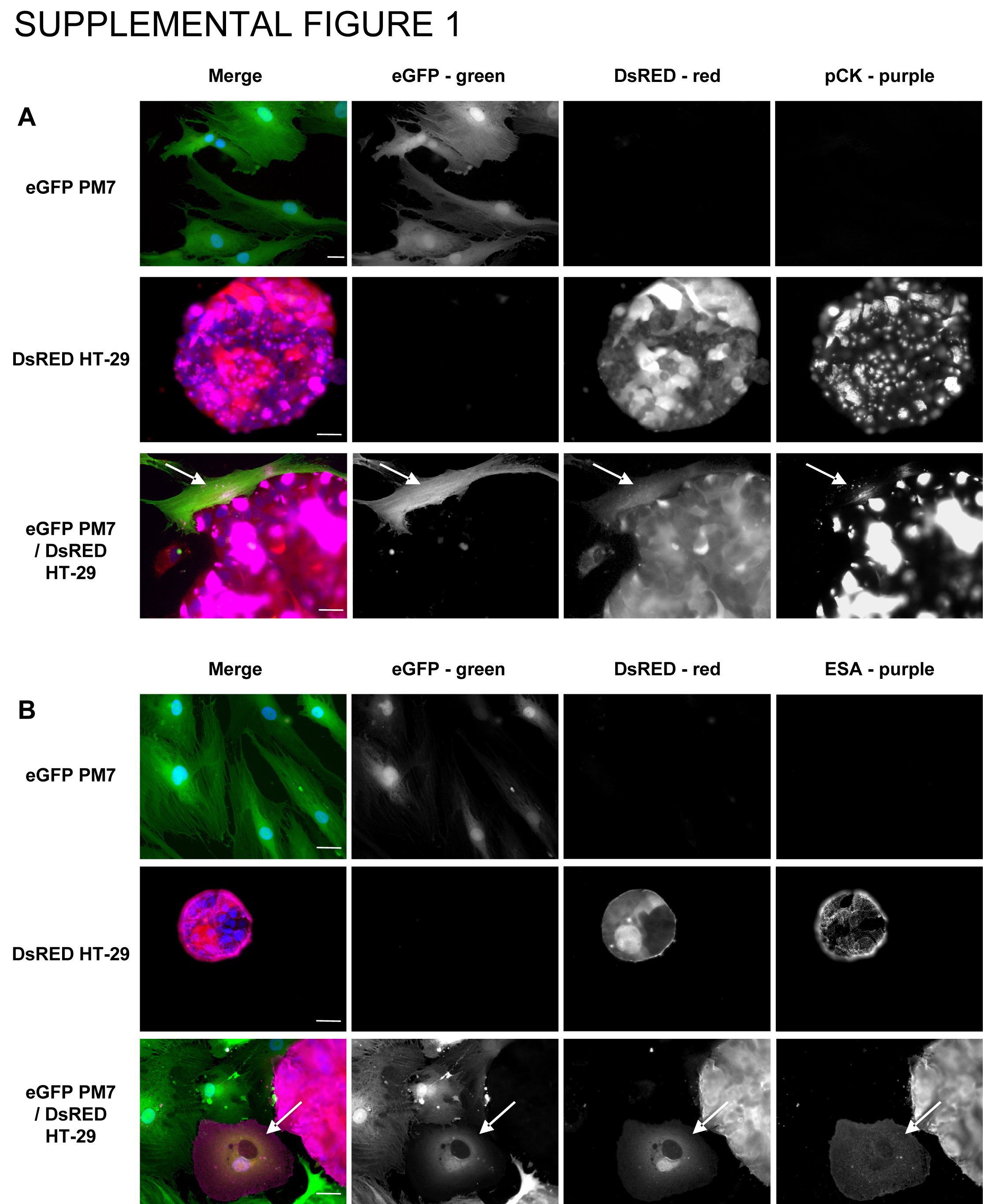

Supplement: Figure S1 — Immunofluorescent staining of epithelial markers in MSC cocultured with colon epithelial cells in vitro . eGFP MSC (PM7) cells were cocultured with DsRED HT-29 epithelial cells for 8 days. A/ Immunostaining with cytokeratins (pCK) or B/ ESA primary antibodies were revealed with AlexaFluor 647 labelled secondary antibodies (purple), and nuclei were stained with Hoechst 33342 compound (blue). The first vertical panel shows colored merge images with Hoechst, whereas black and white channels alone follow. White arrows show MSC fused with epithelial cells and expressing cytokeratins. One representative experiment out of three is presented. Scale bar, 10 µm. (TIF) [file pone.0019569.s001.tif]
